# Gene delivery of a modified antibody to Aβ reduces progression of murine Alzheimer’s disease

**DOI:** 10.1371/journal.pone.0226245

**Published:** 2019-12-30

**Authors:** Bradford M. Elmer, Kurt A. Swanson, Dinesh S. Bangari, Peter A. Piepenhagen, Errin Roberts, Tatyana Taksir, Lei Guo, Maria-Carmen Obinu, Pascal Barneoud, Susan Ryan, Bailin Zhang, Laurent Pradier, Zhi-Yong Yang, Gary J. Nabel

**Affiliations:** 1 Breakthrough Lab, Sanofi, Cambridge, Massachusetts, United States of America; 2 Global Discovery Pathology, Sanofi, Framingham, Massachusetts, United States of America; 3 Translational Sciences, Sanofi, Cambridge, Massachusetts, United States of America; 4 R&D Neuroscience Unit, Sanofi, Chilly-Mazarin, France; Nathan S Kline Institute, UNITED STATES

## Abstract

Antibody therapies for Alzheimer’s Disease (AD) hold promise but have been limited by the inability of these proteins to migrate efficiently across the blood brain barrier (BBB). Central nervous system (CNS) gene transfer by vectors like adeno-associated virus (AAV) overcome this barrier by allowing the bodies’ own cells to produce the therapeutic protein, but previous studies using this method to target amyloid-β have shown success only with truncated single chain antibodies (Abs) lacking an Fc domain. The Fc region mediates effector function and enhances antigen clearance from the brain by neonatal Fc receptor (FcRn)-mediated reverse transcytosis and is therefore desirable to include for such treatments. Here, we show that single chain Abs fused to an Fc domain retaining FcRn binding, but lacking Fc gamma receptor (FcγR) binding, termed a silent scFv-IgG, can be expressed and released into the CNS following gene transfer with AAV. While expression of canonical IgG in the brain led to signs of neurotoxicity, this modified Ab was efficiently secreted from neuronal cells and retained target specificity. Steady state levels in the brain exceeded peak levels obtained by intravenous injection of IgG. AAV-mediated expression of this scFv-IgG reduced cortical and hippocampal plaque load in a transgenic mouse model of progressive β-amyloid plaque accumulation. These findings suggest that CNS gene delivery of a silent anti-Aβ scFv-IgG was well-tolerated, durably expressed and functional in a relevant disease model, demonstrating the potential of this modality for the treatment of Alzheimer’s disease.

## Introduction

Alzheimer’s disease (AD) is characterized by progressive neurodegeneration leading to memory loss and a decline in cognitive function. Its pathological features include the accumulation of extracellular amyloid plaques and intraneuronal tau fibrils. Therapies targeting amyloid beta (Aβ) have been under active investigation for many years due to its genetic and pathologic involvement in AD [1]. While increased levels of amyloid precursor protein (APP) and Aβ are associated with AD pathogenesis, Aβ peptides exist in different conformations and fibrillary status, and it is unclear which species should be targeted for therapeutic benefit [2].

Despite this uncertainty, passive immunotherapy against different forms of Aβ has been extensively tested in the clinic; however, these approaches have been hampered by additional problems. First, at high doses, several anti-Aβ antibodies in clinical trials caused adverse reactions typified by amyloid-related imaging abnormalities (ARIA) thought to be caused by antibody accumulation at sites of vascular amyloid triggering local inflammation via Fc-dependent effector functions [3]. Second, therapeutic antibodies must be given at high concentrations peripherally to overcome the blood brain barrier (BBB) and reach therapeutic levels in the brain.

Gene transfer into the central nervous system (CNS) allows for production of therapeutic protein within neuronal cells and therefore circumvents the BBB. AAV vectors infect both dividing and non-dividing cells, typically exist as stable episomal structures for long term expression and have very low immunogenicity [4]. They are thus well suited for neuronal gene therapy. Transgenes delivered to the brain have been expressed for at least 15 years in non-human primates [5] and 5 years in humans [6], and central delivery may reduce vector neutralization by pre-existing AAV immunity [7, 8].

AAV-mediated expression of either whole immunoglobulins (IgG) or single chain variable fragments (scFv) has been demonstrated within the CNS for various indications [9–15], although both of these approaches have inherent limitations. Heavy and light chain expression of IgG has only been accomplished using a self-cleavable F2A sequence to generate both chains from a single-promoter cassette. The F2A peptide remains attached to either heavy or light chain and is potentially immunogenic and can elicit Abs and/or cellular immunity to the antibody or antibody expressing cells [16]. Gene based delivery of scFv proteins is often accompanied by a substantial loss in affinity due to the loss of valency. Removal of the Fc regions also results in a loss of FcRn binding, causing shorter half-life in the periphery and reduced efflux of antigen-bound scFvs from the brain via reverse transcytosis [17–20]. In contrast, by incorporating an Fc into a scFv-IgG design, the molecule regains the bivalency of canonical IgG providing higher avidity for multimeric targets such as aggregated amyloid, and provides the ability to modulate Fc-dependent signaling if necessary. Preserving Fc-binding to the FcRn at the BBB may improve upon the reduction of amyloid pathology seen previously with scFv alone by enabling antibody-antigen clearance via FcRn mediated efflux from the brain [17–20]. Here, we designed and tested vectors for AAV-mediated expression of anti-amyloid proteins for long-term reduction of amyloid pathology in the brain.

## Results

### Construction and characterization of an AAV-IgG vector targeting β-amyloid

**Fig 1:**
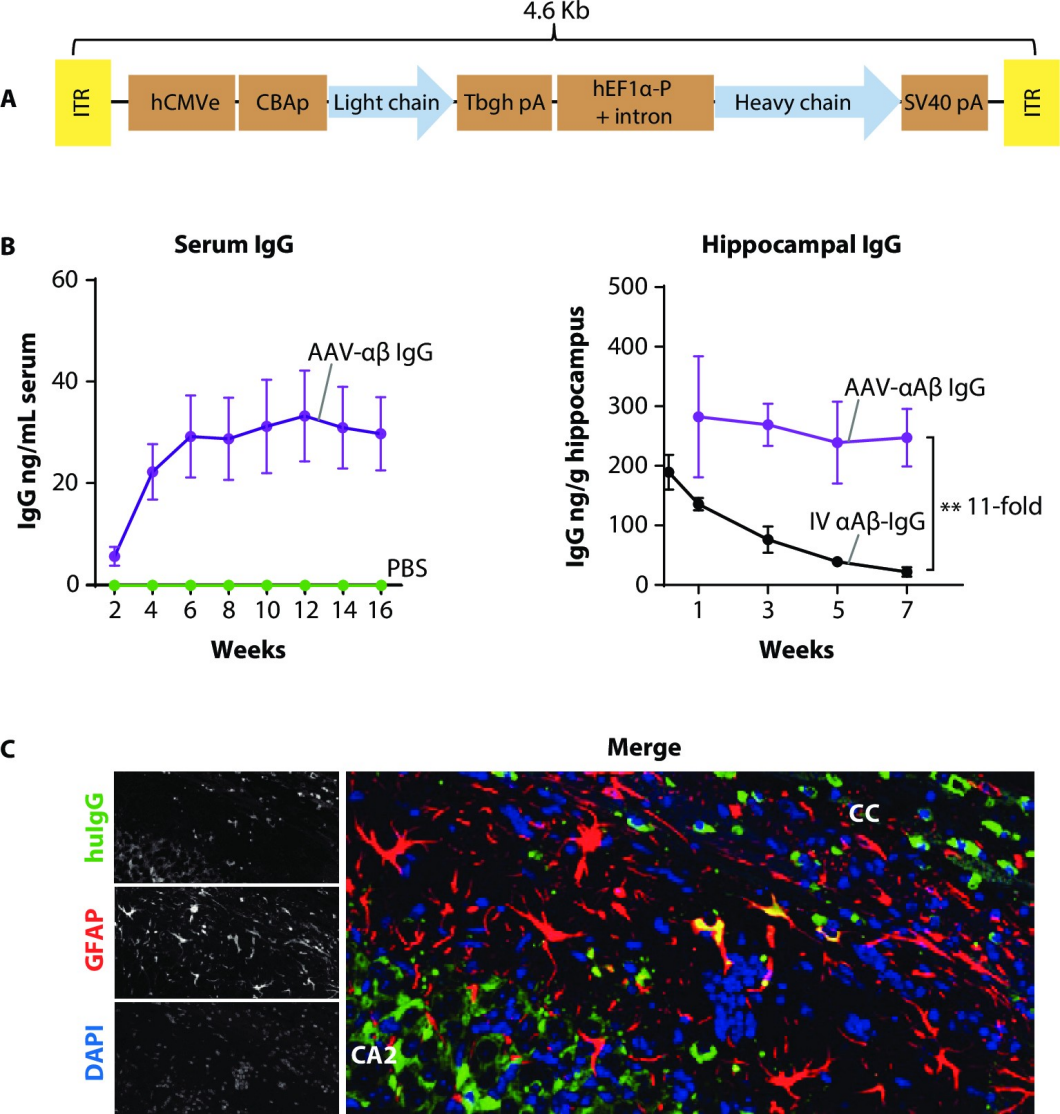
Construction and characterization of an AAV-IgG vector. A) A schematic of the dual promoter cassette for full heavy and light chain expression. The size of the genome is indicated. B) Graph on left shows durable expression and secretion of AAV-αAβ IgG from the brain. Sera was drawn at 2 week intervals for 16 weeks following bilateral injection of AAV-αAβ IgG into the hippocampus (2E10 GC per side) of SCID mice. Purple line is the AAV-αAβ IgG expression level. Green line is huIgG measured from PBS injected control mice. Graphed points represent the mean +/- SEM, n = 8 mice per group. Graph at right shows the dynamics of AAV-mediated expression of AAV-αAβ IgG in the brain versus traditional peripherally administered αAβ IgG. SCID mice were injected once with 2E10 GC of AAV- αAβ IgG bilaterally into the hippocampus, or once with 20mg/kg IV purified IgG before tissue collection at the indicated times to generate a time course of brain exposure to IgG. Ipsilateral hippocampi were homogenized and assayed for huIgG by antigen ELISA. Graphs show the mean +/- SEM. **p<0.01, 1-way ANOVA at 7 weeks post injection, n = 5 mice per time point. C) Intraneuronal and glial expression of AAV-IgG is detectable in the hippocampus. Micrograph shows neurons expressing the huIgG transgene in neurons throughout the hippocampus (CA2 shown in detail), with some GFAP+ astrocytes nearby also expressing huIgG. Cc = corpus callosum.

To develop a single vector for gene-based expression of a full-length antibody, we used a dual promoter expression cassette encoding a humanized version of an anti-β-amyloid antibody that binds protofibrillar and fibrillar Aβ with no affinity for monomeric forms [21]. The IgG heavy and light chains were expressed from different promoters, and the entire cassette was designed to fit within the AAV genome packaging limit of approximately 4.6 kilobases (Fig 1A). The IgG4 isotype heavy chain included mutations designed to reduce Fcγ effector function, and half-molecule exchange to improve stability [22, 23]. Expression of heavy and light chain proteins from separate promoters reduces potential immunogenic or expression liabilities induced by other single-promoter designs that require the use of self-cleaving sequences or internal ribosomal entry site for bicistronic expression [16, 24]. This cassette was packaged into an AAV1 capsid (AAV-αAβ IgG) for direct injection into the brain because this serotype exhibits excellent spread within brain tissue and can transduce astrocytes, which may be more amenable to high level protein expression and secretion than neurons. C57BL/6-SCID (SCID) mice were used to test for in vivo expression of the vector to prevent anti-huIgG immune responses that could interfere with expression of the transgene. IgG is actively transported out of the brain via reverse transcytosis, therefore we monitored brain expression of the AAV-αAβ IgG using biweekly serum collection. An Aβ_1–42_ fibril binding immunoassay (antigen ELISA) was used to measure levels of expressed, functional antibody following bilateral hippocampal injection of 2E10 genome copies (GC) of AAV-αAβ IgG. The vector demonstrated stable expression for up to 16 weeks (Fig 1B left). To gain insight into how AAV-mediated antibody expression in the brain compares to levels observed following a standard passive immunotherapy approach, huIgG levels in the hippocampus of SCID mice were measured at different time points in parallel with a separate group that received a single intravenous (IV) bolus injection of 20mg/kg αAβ huIgG. The AAV-αAβ IgG vector sustained expression of huIgG in the hippocampus at almost 300ng/g for the duration of the time course as measured by antigen ELISA (Fig 1B right). Levels of IgG in the hippocampus 24 hours after IV injection approached 200ng/g, but these levels declined as the IgG was cleared from the brain (in line with known serum half-life), resulting in a 11-fold reduction compared to the AAV-αAβ IgG by 7 weeks. Expression in both neurons and astrocytes was confirmed by immunohistochemistry (IHC) against the huIgG expression product, with neurons readily identifiable via morphology in CA2 of the hippocampus, and colocalization with the glial fibrillary acidic protein (GFAP) indicating astrocytic expression (Fig 1C). These data show that the AAV-αAβ IgG vector can maintain steady-state levels of antibody in the brain significantly higher than that which can be achieved by traditional passive immunotherapy protocols.

### Antigen binding by AAV-αAβ IgG in a mouse model of Alzheimer’s disease

**Fig 2:**
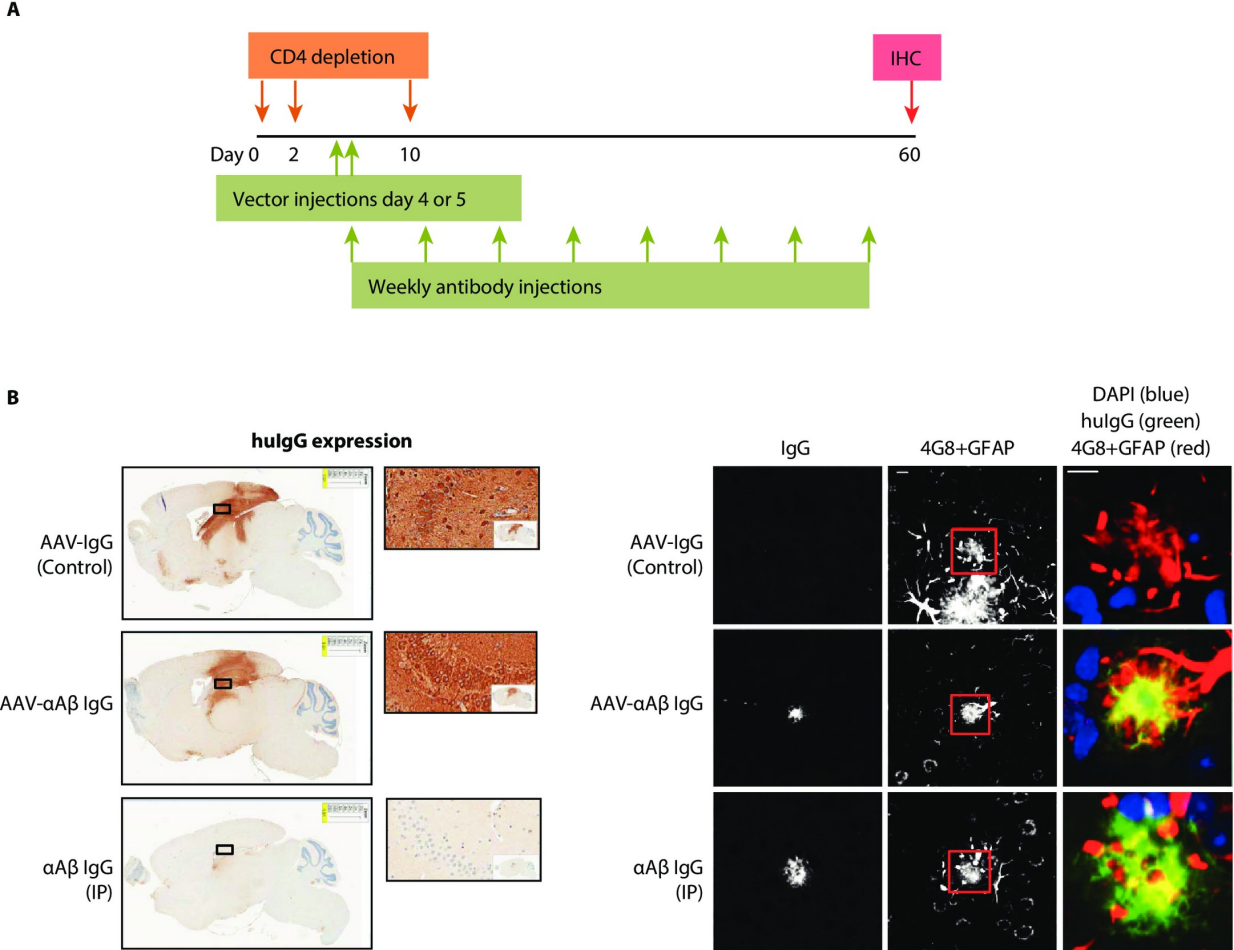
Antigen binding by AAV-αAβ IgG in a mouse model of amyloid plaque deposition. A) Study design for intracranial (AAV-αAβ IgG or AAV-IgG Control) and peripheral dosing (αAβ IgG). APP mice were immunotolerized by CD4 T-cell depletion between days 2–10. AAV-αAβ IgG, or the isotype control vector AAV-IgG Control, were injected into the hippocampus bilaterally (2E10 GC per injection) at days 4–5. Purified αAβ huIgG was injected weekly, IP at 10mg/kg. After 8 weeks, 5um sagittal brain sections were collected and immunostained as indicated below. B) Expression of AAV-αAβ IgG or AAV-IgG Control. huIgG IHC revealed expression throughout the hippocampus and overlying cortex. Magnified ROIs (500um width) show detail of huIgG expression in neurons and in the neuropil of the hippocampus. Animals receiving antibody injected IP did not exhibit any expression in cell bodies. Images at right show IgG binding to plaques in frontal cortex. AAV-αAβ IgG and peripherally delivered αAβ IgG displayed clear binding to 4G8+ amyloid deposits, while the AAV-IgG Control did not display detectable binding. Scale bars are 10um.

We next expressed the AAV-αAβ IgG in an amyloid plaque mouse model that expresses mutant amyloid precursor protein (APP mice) to assess the extent of brain transduction and determine whether the antibody is secreted into the extracellular space to bind amyloid plaques in vivo. This model exhibits progressive amyloid plaque accumulation in the hippocampus and cortex starting around 2–3 months of age [25]. To readily detect the IgG in mice, we injected 2 month old, male APP mice intra-hippocampally with AAV-αAβ IgG or an AAV expressing an isotype control IgG (AAV-IgG Control). A separate group was injected IP weekly with purified αAβ IgG protein (the same antibody expressed from our AAV) at 10mg/kg for the duration of the study as a positive control for plaque binding activity (Fig 2A). To prevent anti-huIgG antibody responses, animals were immunotolerized with a CD4-depleting antibody before and after vector administration (Fig 2A). Importantly, CD4 depletion in this mouse model has no effect on baseline amyloid deposition or efficacy of anti-Aβ passive immunotherapy [26, 27]. Two months after injection, at an age where these animals exhibit plaque deposition in frontal cortex, sagittal sections of brain were processed for anti-huIgG IHC. In contrast to the IP αAβ IgG group, with staining limited to amyloid plaques, AAV-αAβ IgG and AAV-IgG Control groups showed transgene expression throughout the entire hippocampus and overlying cortex surrounding the needle track (Fig 2B left). Fluorescence IHC for huIgG, Aβ plaques and GFAP showed colocalization of huIgG with cortical plaques in both the AAV-αAβ IgG and the IV αAβ IgG groups, but not in the AAV-IgG control group (Fig 2B right). These data show that the AAV-αAβ IgG is secreted into the extracellular space and can bind to Aβ plaques in brain regions distal to the site of injection.

### Evaluation of AAV-αAβ IgG neuronal expression and neurotoxicity

**Fig 3:**
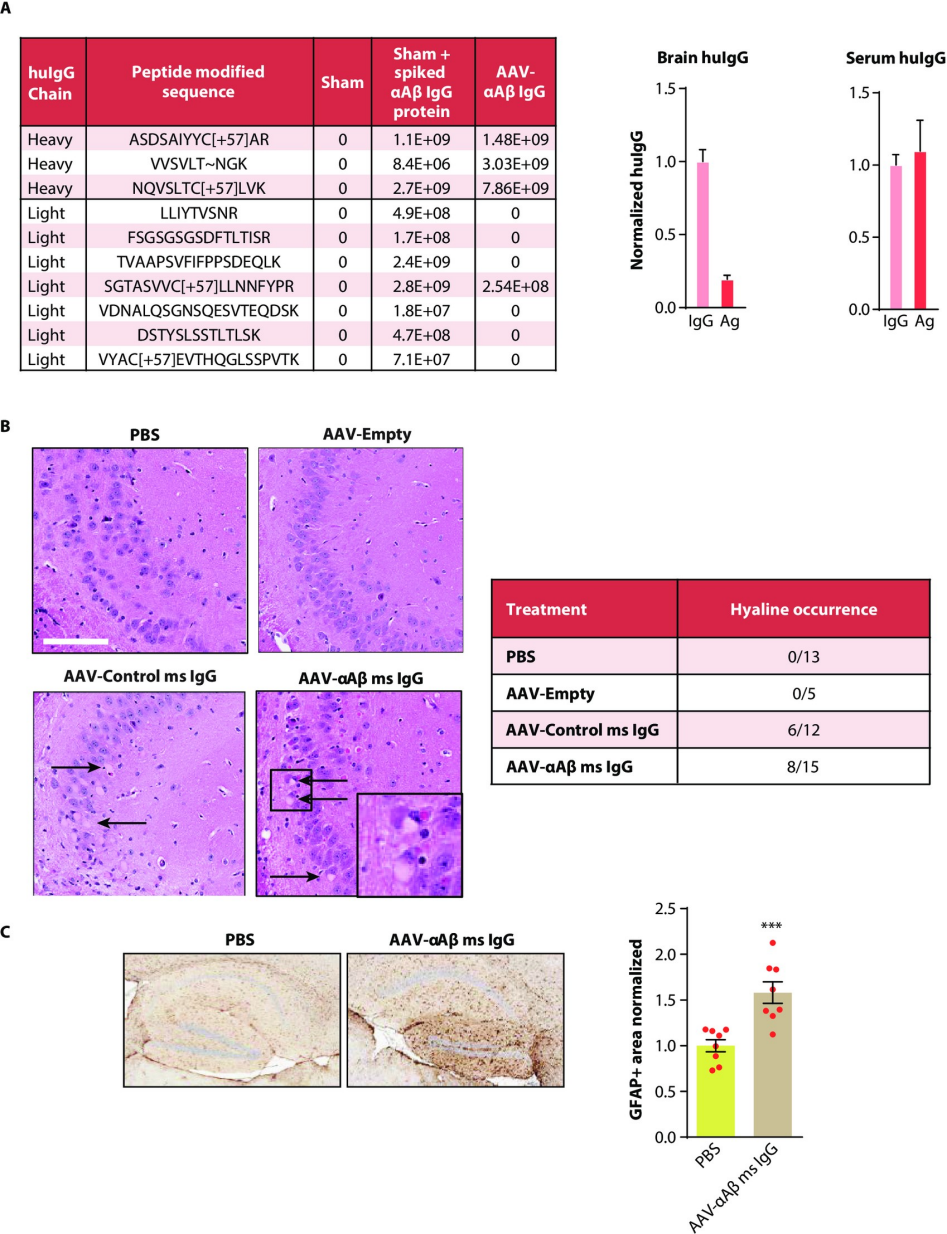
Evaluation of AAV-αAβ IgG neuronal expression and neurotoxicity. A) LCMSMS analysis showing detected peptides from huIgG heavy and light chain from hemibrain lysates of SCID mice injected with AAV-αAβ IgG compared to animals injected with PBS (Sham), or Sham brain homogenate spiked with equivalent levels of huIgG as in the AAV-αAβ IgG group. Graphs at right show quantification of functional huIgG compared to total huIgG expressed in SCID mice either centrally (2E10 total GC injected into hippocampus) or peripherally (1E12 total GC injected IV). Levels of functional Aβ antibody in brain extracts from AAV-αAβ IgG expressing SCID mice were quantified by antigen (Ag) ELISA and compared in parallel with a pan-huIgG ELISA (IgG). Levels of mean huIgG bound to antigen accounted for only 21% of mean total huIgG when expressed from the brain whereas this discrepancy is not detected in sera one month following peripheral expression of the vector. Data are presented as mean +/- SEM. **p<0.01, unpaired Students t-test. B) H&E staining of C57BL/6 mouse brain hippocampus following intra-hippocampal injection of the indicated vectors compared to PBS control. H&E staining revealed neuronal, eosinophilic to hyaline-like inclusions reminiscent of glycoprotein accumulation only in brains injected with the antibody expression vectors, while PBS and AAV-Empty vector injected animals did not have this pathology. Inset shows detail, arrows point to representative hyaline inclusions. Scale bar = 100μm. Results are summarized in the table at right as the number of animals scored with or without this pathology. C) Quantitative IHC for GFAP+ area. C57BL/6 mice were injected with either PBS or AAV-αAβ msIgG (2E10 GC into hippocampus), and 5μm sagittal brain sections were collected 16 weeks later. Each circle represents one mouse. Bars indicate group mean +/- SEM of GFAP+ area normalized to PBS. ***p<0.001, unpaired Students t-test, n = 8 mice per group.

Neuronal cells are highly specialized to secrete factors relevant to neurotransmission rather than large macromolecules such as IgG. Whether efficient IgG processing and secretion can occur in these cells is unknown. To determine whether there was improper processing of the neuronally-expressed IgG, we performed mass spectrometry analysis to measure overall levels of heavy and light chains from brains after 1 month of AAV-αAβ IgG expression in SCID mice. Expression of the AAV-αAβ IgG from the hippocampus was associated with expected levels of heavy chain–similar to saline injected brain lysates spiked with purified αAβ IgG, but an unexpectedly low amount of cognate light chain when compared to the spiked control (Fig 3A). This finding suggested that AAV-αAβ IgG expression from brain cells resulted in insufficient light chain production resulting in an imbalance in the proportion of heavy and light chains. We also used enzyme linked immunosorbent assays (ELISAs) to quantify total IgG (H+L chains) vs the percent of that population that can bind antigen (Ag). We observed that 21% of the mean total IgG expressed from the brain was functional, while AAV-αAβ IgG expressed from peripheral tissues via an IV injection of vector did not have an imbalance in total IgG/functional IgG (Fig 3A right graph). We next investigated whether there was evidence for neurotoxicity as a result of IgG expression. For our initial characterizations of the AAV-αAβ IgG vector, we had used the huIgG version of this antibody which has more direct translational potential for humans and allowed for clear detection in mice. However, to test for any toxicity or neuroinflammation that could be related to brain IgG expression without the confounding variable of xenogenic huIgG exposure, we used an AAV vector -termed AAV-αAβ msIgG- expressing the original mouse version of the αAβ IgG [21, 26]. This vector was injected into the hippocampus of C57BL/6 mice and brain tissue was processed for histology one month later. Histopathological analysis revealed a high incidence of hyaline/eosinophilic cytoplasmic deposits in neuronal cells in the hippocampus–reminiscent of glycoprotein overexpression (Fig 3B). These structures were also observed in the hippocampus of mice injected with the AAV-IgG Control (6/12 mice) indicating that this toxicity was not specific to αAβ IgG expression. These hyaline deposits were never observed in the hippocampus of mice injected with an AAV-Empty vector, or PBS alone (Fig 3B). We also observed evidence of neuroinflammation by immunohistochemical GFAP analysis relative to PBS (Fig 3C). AAV-Empty vector also did not elicit significant gliosis relative to PBS (1.11+/-0.12, 5 mice, mean+/-SEM normalized to PBS GFAP+ area), suggesting that the neuroinflammation is due to IgG expression. These data indicate that while brain cells can express and secrete whole IgG, only a subset of this IgG is functional and can bind antigen, and this expression induces detectable neuroinflammation throughout the transduced region.

### Construction and characterization of an AAV-scFv-IgG vector

**Fig 4:**
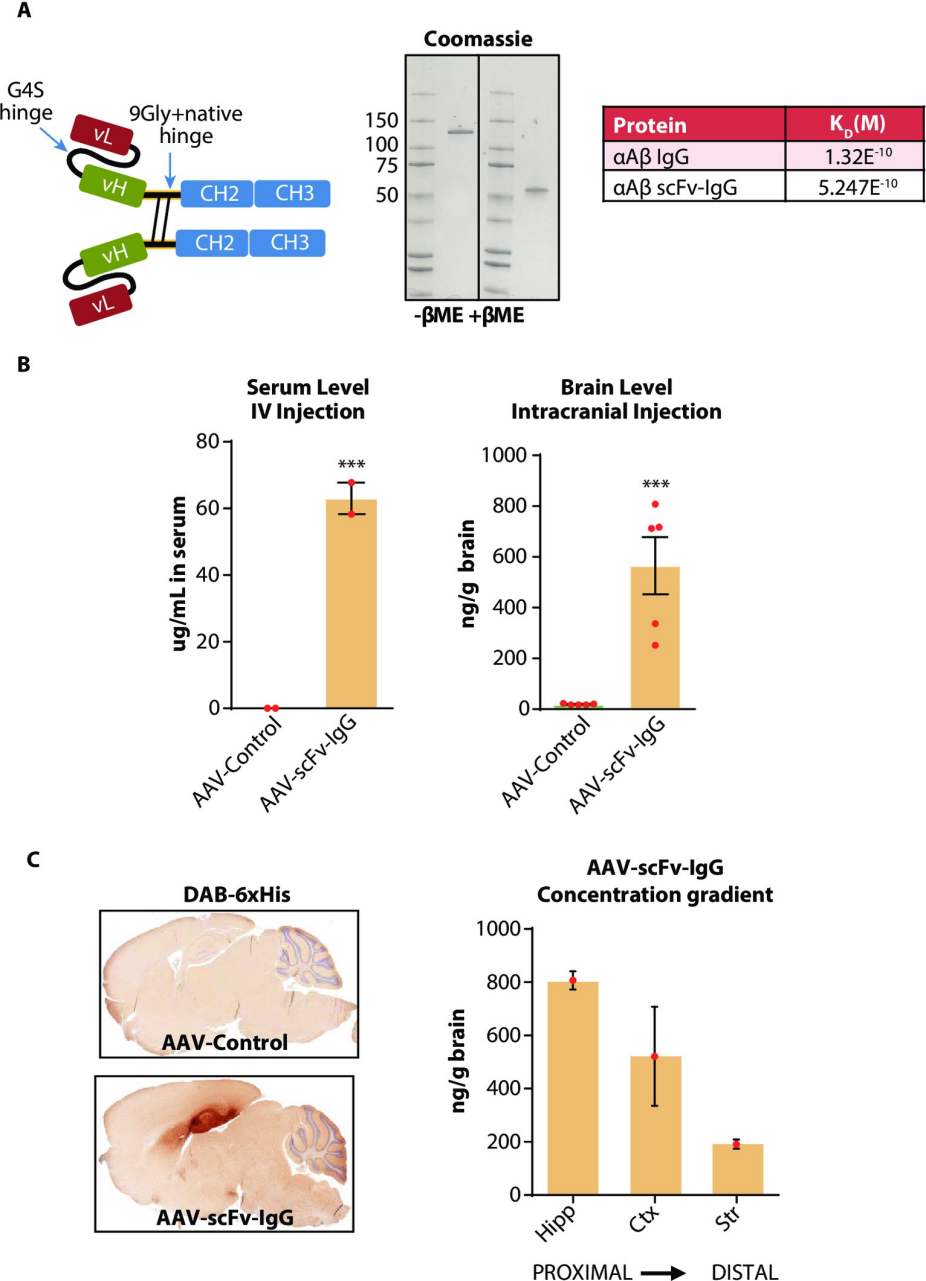
Construction and characterization of an AAV-scFv-IgG vector. A) Schematic of the scFv-IgG design. The scFv-IgG used the variable regions of the murine anti-Aβ IgG [21] linked via 3 repeats of a flexible GGGGS linker sequence. The scFv was linked to the mouse IgG1 N297A Fc via a 9-Gly repeat linker. A 6xHis tag was added to the C-terminus. Reducing or non-reducing SDS-PAGE analysis of the purified scFv-IgG demonstrated purity and proper disulfide-dependent dimerization of the protein. Table at right compares antigen binding affinity of the scFv-IgG versus the IgG format. Affinity (M) was determined via SPR by flowing the scFv-IgG or IgG over immobilized Aβ_1–42_ fibrils at different molar concentrations to analyze binding kinetics. B) Left hand graph shows serum expression of the AAV-scFv-IgG as measured by antigen ELISA one month following peripheral injections of AAV into C57BL/6 mice. Vector was delivered by IV injection of 1E12 total GC. Right hand graph shows brain expression of the AAV-scFv-IgG as measured by antigen ELISA 1 month following hippocampal injection of 2E10 total GC of AAV into C57BL6 mice. ***p<0.001, unpaired Students t-test, n = 5 mice per group for intracranial injection, 2 mice per group for IV injection. **C**) DAB-6xHis immunohistochemistry on sagittal sections of mouse brain taken from the same animals as in (B, right) demonstrating hippocampal targeting of the vector, and transduction throughout the hippocampal formation. Graph to the right shows ELISA-based quantification of scFv-IgG in different brain regions after bilateral hippocampal injection of 1E10 GC of AAV-scFv-IgG. One month following AAV injection, brain regions from 3 mice were dissected and expressed protein was quantified by antigen ELISA for each brain region, with PBS-injected brain homogenate used to subtract background signal. Hipp = hippocampus. Ctx = overlying cortical regions. Str = striatum.

While the IgG delivered by our vector was secreted and bound amyloid plaques in vivo, we hypothesized that an alternate Ig format could minimize the mispairing and neurotoxicity induced by expression of the whole IgGs. Based on the same mouse αAβ antibody [21], we synthesized a modified single chain Fv, with the variable region of the IgG light chain fused to the heavy chain variable region which was connected by the COOH-terminus (C-terminus) to the murine IgG1 hinge, CH2 and CH3 domains, similar to that shown in [28] (Fig 4A; scFv-IgG). To minimize the pro-inflammatory effects of the Fc region, the mouse IgG1 Fc domain was mutated to eliminate glycosylation at asparagine 297 (N297A) which prevents binding to all FcγRs [29, 30]. The scFv-IgG was expressed in Expi293 cells and purified by immobilized metal affinity chromatography (IMAC) using a C-terminal histidine (His) tag sequence. Analysis by SDS-PAGE confirmed that this protein efficiently assembled into a disulfide-linked dimer (Fig 4A). Binding to fibrillar Aβ_1–42_ was measured by surface plasmon resonance (SPR) and this scFv-IgG protein displayed binding comparable to the parental antibody. The parental IgG exhibited an apparent dissociation constant (K_D_) of 1.3E^-10^ M compared to a slightly lower binding affinity of 5.2E^-10^ for the scFv-IgG (Fig 4A Table).

This expression cassette was inserted into an AAV1 vector to determine whether the modified IgG could be synthesized in vivo. IV injection of the AAV was used as a positive control for activity of our virus as peripheral tissues are well validated for expression and secretion of IgG molecules [16, 31–36]. One month after IV injection of AAV-scFv-IgG, serum levels reached 63μg/mL, demonstrating robust AAV vector activity in peripheral tissues (Fig 4B left). To assess brain expression of the vector, scFv-IgG levels were quantified from extracts derived from one sagittal half of the brain, termed hemibrain, one month after hippocampal injection. Expression levels reached a mean of ~600ng/g (Fig 4B right). Notably, this concentration is >3-fold higher than that observed 24 hrs after a 20mg/kg IV injection of IgG, and 2.5-fold higher than that observed by AAV-αAβ IgG (Fig 1B). Histological analysis revealed that despite having higher levels of expression in the brain than the AAV-αAβ IgG vector, AAV-scFv-IgG transduction did not cause any detectable intraneuronal hyaline protein accumulation in the injected hippocampus (0/5 mice), like the PBS control image shown in Fig 3B, suggesting that the scFv-IgG is more effectively processed by neuronal cells than the IgG.

To define the brain distribution of scFv-IgG transduced cells, IHC was performed on sagittal sections one month after hippocampal injection using an antibody to the His tag. The AAV-scFv-IgG vector transduced the entire hippocampus, with sparse transduction in the cortical area overlying the hippocampus around the needle track and subiculum (Fig 4C). Brains transduced with negative control, empty AAV (AAV-Control), vectors did not show detectable anti-His immunostaining (Fig 4C). It should be noted that anti-His IHC in C57BL6 mice only detects intracellular expression, as any secreted, extracellular scFc-IgG is likely washed away due to a lack of available antigen. Expression of both intracellular and extracellular scFv-IgG was evaluated biochemically in ipsilateral brain regions both proximal and distal to the site of injection. Hippocampus, overlying cortex, and striatum were dissected and homogenized for quantification of scFv-IgG via antigen ELISA. A concentration gradient was observed, with highest levels detected in the injection site (hippocampus), and progressively lower levels observed in more distal brain regions (Fig 4C right). Despite having lower levels compared to the injection site, the concentration of the scFv-IgG in striatal tissue remained near 200ng/g–steady state levels in the brain not typically attained by passive IgG infusion.

### Antigen binding by scFv-IgG in a mouse model of β-amyloidosis

**Fig 5:**
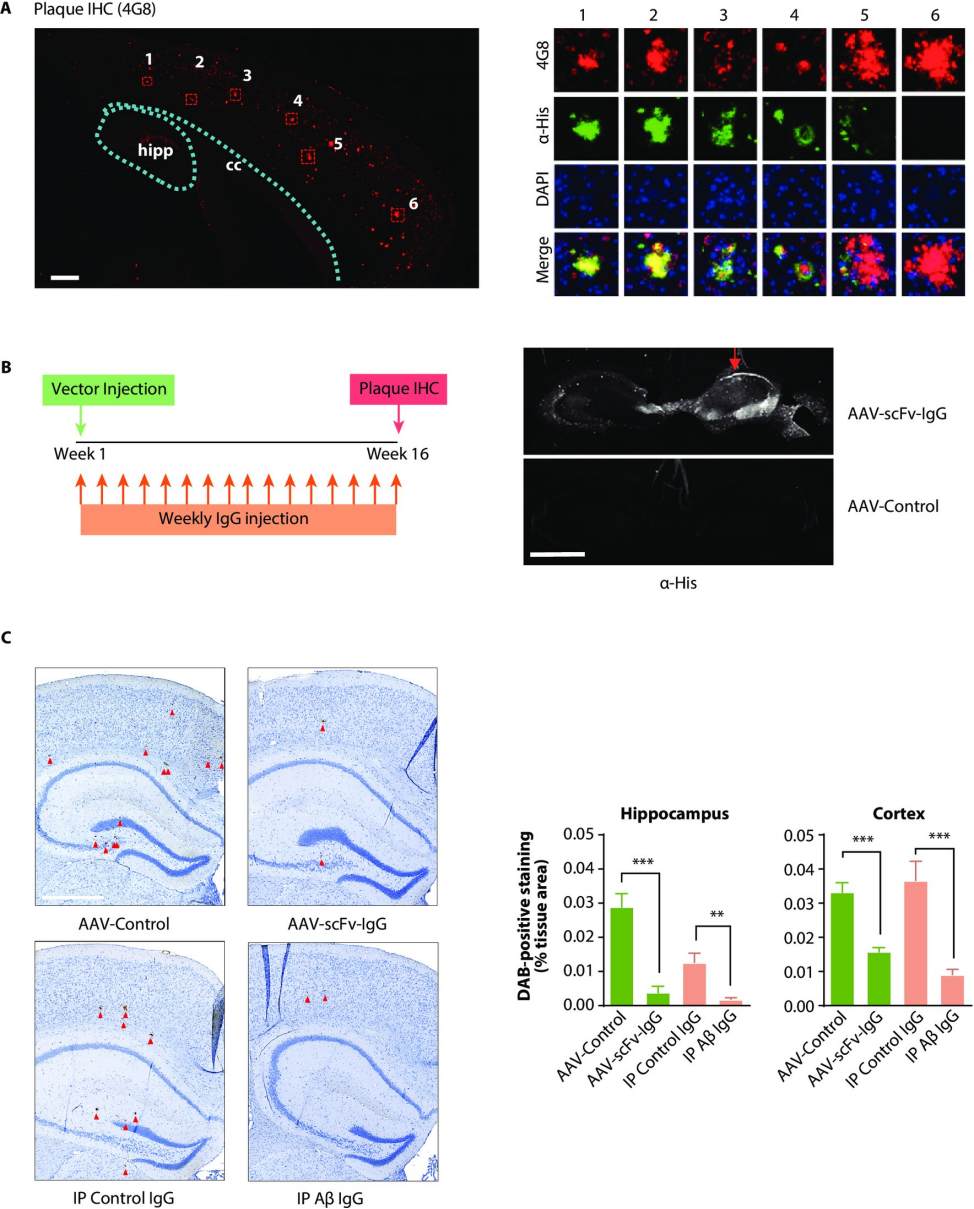
Expression, diffusion, and plaque binding of the anti-Aβ scFv-IgG. Five month old female APP animals with pre-existing plaque pathology were injected unilaterally with 1μL (1E10 total GC) of AAV-scFv-IgG vector, and 5um sagittal sections of brain were collected 1 month later. A) Whole scan of hippocampus and overlying cortex of 5 month old APP mice 1 month post-injection with anti-Aβ AAV-scFv-IgG. Sections were immunostained for amyloid beta plaques (4G8, red) and 6xHis (green). Scale bar = 300μm. Cc = corpus callosum. Images at right show individual plaque ROIs (numbered in A) proximal (1) to distal (6) from the site of injection. Images were overlaid with 6xHis immunostaining (green) and DAPI (blue). Note the progressive but far reaching reduction in the intensity of 6xHis labeling on plaques that are more distal to the hippocampus and occipital cortical areas of AAV-scFv-IgG expression. ROIs are 150μm in diameter. B) Outline of study design. Four groups of 2 month old APP male mice were injected with either the AAV-scFv-IgG, AAV-Control vector, Aβ IgG, or control isotype IgG. The AAV vectors were injected unilaterally into the hippocampus, while IgGs were injected IP weekly at 10mg/kg for 16 weeks. Brains were collected after 4 months of treatment, and coronal sections of brain were immunostained for amyloid plaques or 6xHis. Images to the right show the hippocampus from coronal sections of AAV-injected mice. Anti-His IHC revealed labeling throughout the hippocampus on the injected side (red arrow), with additional transduction of the contralateral hippocampus. AAV-empty injected brain did not show any anti-His labeling. Scale bar = 1mm. **C**) Representative images of coronal brain sections immunostained and enlarged to show cortical and hippocampal 4G8^+^ amyloid plaques (dark brown stain). Both control groups showed clear plaque accumulation in cortex and hippocampus, whereas animals treated with the AAV-scFv-Fc targeting Aβ, or the positive control Aβ IgG had significantly fewer plaques. Plaques are indicated by red arrowheads. The scale bar is 500μm. The graph to the right shows the quantification of plaque deposition in cortex and hippocampus of animals from each respective group. ROIs from both hemispheres were combined for quantification and plaque load is expressed as DAB-positive staining as a percent of tissue ROI area. n = 10–13 animals per group, 3 sections per animal. ***p<0.001 one-way ANOVA with multiple comparisons. Errors bars represent S.E.M.

We next determined whether AAV delivered scFv-IgG was secreted into the extracellular space and could bind to antigen *in vivo*. The AAV-scFv-IgG vector was injected into the hippocampus of 5 month old female APP mice [25], an age when they have already developed plaques throughout the neocortex. Sagittal sections of brains were processed for IHC one month after injection and stained for His tag reactivity and Aβ plaques. As expected, abundant plaque formation was observed throughout the cortex (Fig 5A, left) and staining with an anti-His antibody co-localized with plaques (Fig 5A, right). Importantly, there was a clear concentration gradient of plaque-bound scFv-IgG, with plaques distal to the hippocampus showing progressively lower levels of bound scFv-IgG than plaques closer to the site of injection. These data indicate that the anti-Aβ scFv-IgG is expressed and secreted from cells in the hippocampus, which allows it to bind to plaques distal to the injection site.

These data provided evidence that anti-Aβ scFv-IgG delivered by the viral vector engaged its physiologically relevant target *in vivo*. We next determined whether long-term expression in this mouse model of amyloidosis might reduce plaque formation. For this study, the mice were injected at an age when plaques were just beginning to form. Separate groups of two month old male APP mice were injected unilaterally into the hippocampus with either the AAV-scFv-IgG or negative control AAV empty vector. These groups were compared to animals treated with passive immunotherapy with weekly IP injections of the mouse anti-Aβ antibody (Aβ IgG) or an isotype control antibody at 10mg/kg (Fig 5B, left). Brains were collected after 16 weeks of treatment and coronal sections were analyzed for transgene expression. The AAV-scFv-IgG was expressed throughout the injected hippocampus, and there was also clear transport of vector into the contralateral subiculum, as evidenced by αHis staining of cell bodies (Fig 5B, right). Aβ plaque load in cortex and hippocampus was quantified by IHC in coronal brain sections (Fig 5C, left). Compared to their respective controls, a single injection of the AAV-scFv-IgG caused the same magnitude of plaque reduction in the hippocampus as the αAβ IgG benchmark, despite the differences in plaque load between the control groups (Fig 5C, graph). Plaque reduction was also significantly reduced in cortex (Fig 5C graph), consistent with evidence that the scFv-IgG diffuses from the site of expression to bind to distal plaques.

These results demonstrated that a single injection of the AAV-scFv-IgG in an amyloid mouse model is durably expressed and secreted from the site of injection to bind to plaques throughout the brain. The typical passive immunotherapy regimen of 10mg/kg weekly anti-Aβ IgG for 16 weeks caused significant reductions in amyloid plaque formation in APP animals. In contrast, a single intracranial injection of the AAV-scFc-IgG resulted in comparable efficacy after 4 months of expression.

## Discussion

Passive transfer of antibodies against Aβ have been shown to modify the pathology of Alzheimer’s disease in animal models, but translation to the clinic has proven challenging [37]. The lack of efficacy of Ab therapy in clinical trials has been attributed to several factors. Principal among them is that patients were recruited too late in the course of the disease to provide substantial cognitive benefits [37]. Treatment with these antibodies has been subject to two additional limitations. First, the blood brain barrier restricts transport of large biomolecules, necessitating the injection of high doses in the periphery to reach therapeutically relevant levels in the brain. Second, there is the need to maintain levels above a minimal therapeutic dose, requiring long-term passive immunotherapy that requires patient engagement and compliance, as well as a significant cost of goods.

Immunotherapy with AAV vectors addresses these problems in several ways. These vectors are ideal for CNS gene delivery because they infect both dividing and non-dividing cells, exist as stable episomal structures for long term expression, and have very low immunogenicity [7, 8]. However AAV-mediated expression of whole immunoglobulins or single chain variable fragments (scFv) in the brain comes with inherent limitations. Neuronal cells are highly specialized to secrete factors relevant to neurotransmission rather than large macromolecules such as IgG. Whether efficient processing and secretion can occur in the absence of natural chaperone proteins in vivo was unknown. Indeed, in this study, expression of IgG using a dual promoter system for separate heavy and light chain production in hippocampal neurons in vivo resulted in inadequate pairing of heavy and light chains, glycoprotein accumulation in neurons, with low levels of functional, antigen-binding IgG molecules. This expression was also associated with detectable gliosis–a phenomenon reported by others who have expressed whole IgG directly in the brain by AAV [15]. While the smaller scFv fragments have been packaged and expressed in the CNS with AAV vectors [9–14], these molecules are monovalent rather than bivalent, leading to a lower binding affinity. In addition, the lack of a Fc domain on scFv fragments eliminates FcRn binding that is important for efflux of antigen:IgG complex from the brain via reverse transcytosis [17–19]. ScFv fragments are also typically more prone to aggregation and poor expression, complicating gene delivery efforts, however conjugation to a larger molecule such as an IgG Fc can improve stability and resolve this issue [38, 39]. The scFv-IgG format therefore has several attractive features desirable for the delivery of therapeutic Abs to the CNS.

In this study, the scFv-IgG was derived from an antibody specific for protofibrillar and fibrillar Aβ species that reduces amyloid plaque load in vivo [21, 40]. These Aβ species are particularly toxic for neurons [41, 42], and their reduction mitigates Aβ-dependent synaptotoxicity, resulting in improved synaptic function. While plaque reduction was used as the benchmark for efficacy in the current study, it is also possible that therapeutic benefits could be derived due to the sequestration or masking of synaptotoxic, soluble Aβ species by our scFv-IgG. This is consistent with recent data demonstrating that the parental antibody our molecule is derived from blocks Aβ42 oligomer-induced neurotoxicity in primary neuronal cultures and prevents synaptic activity deficits in vivo[27]. The modular nature of the scFv-IgG means this format could be used to express other anti-Aβ antibodies, for example, those that may have improved or selective recognition of synaptotoxic species that may be isolated in the future. Additionally, using an Fc with reduced effector function allows for a higher safety margin for dosing by avoiding side-effects related to vasogenic edema/ARIA [37]. Our scFv-IgG expressed well in vitro, allowing for purification and subsequent analysis of antigen binding affinity by SPR. Compared to the IgG form, the scFv-IgG bound antigen to a similar extent, with only a minor reduction in K_D_.

AAV1 was chosen as the serotype for this indication because its capsid facilitates vector spread in the CNS following parenchymal injection. This serotype infects predominantly neuronal cells, but does transduce some non-neuronal cell types, expanding the potential repertoire of cells available for transgene expression. Using relatively high dosing (1E10 GC in the hippocampus), steady state levels at the site of injection is 3–4 times higher than what could be maximally achieved with the passive IgG benchmark we chose for comparison (antibody levels in the brain 24hrs post-20mg/kg IV injection of purified IgG). Dosing in the periphery of ~60mg/kg IV would be needed to reach the levels attained by the AAV-scFv-IgG vector.

Following a single injection, expression in the hippocampus was sustained for at least 4 months, with protein concentrations exceeding the passive immunotherapy benchmark even in brain regions several millimeters distal to the injection site. It is unlikely that transduced cells migrate from the site of injection to secrete protein in regions distal to the hippocampus, as 6xHis positive cells were not observed near plaques far beyond the site of injection or needle tract (Fig 5A right). Long term expression of this vector in APP mice caused plaque reduction both in the cortex (52% reduction) and hippocampus (87% reduction). This is a more efficient reduction than that observed by other studies utilizing scFvs, where plaque reduction ranged between 0–60% [11–14, 43]. This difference is notable and may be due to the different IgG variable regions used, different serotypes, amyloid models, expression levels, and durations of treatment. The contralateral hippocampus of injected animals did have sparsely transduced cells, indicative of AAV transport across the hippocampal commissure, which would also increase levels of scFv-IgG in the uninjected side of the brain and highlights the utility of harnessing active transport to deliver vector to distal brain regions. The observed magnitude of plaque reduction in animals treated with a single intracranial injection of AAV-scFv-IgG was similar to animals treated with weekly IV injections of 10mg/kg anti-Aβ antibody for 4 months, highlighting the value of gene delivery for long term treatment paradigms.

It is important to highlight that no mouse model can faithfully replicate human Alzheimer’s disease pathogenesis. Transgenic APP mouse models like those used here are widely used for preclinical testing of therapies for AD, but they do not recapitulate key components of the disease, such as tau neuropathology [25, 27]. These mice do have an age-dependent, progressive accumulation of amyloid plaques in brain regions relevant to AD, providing a valuable in vivo model for rapidly testing amyloid lowering strategies for AD.

AAV-scFv-IgG delivery to the brain provides multiple advantages over traditional, passive IgG immunotherapy. Accumulating evidence suggests that prophylactic treatments will be required to enable disease modification in neurodegenerative diseases, necessitating decades of treatment [37]. Delivery of AAV vectors to the brain parenchyma is well established in non-human primates and humans, with expression in the brain documented for at least 15 years in non-human primates [5] and more than 6 years in patients injected with an AAV vector encoding nerve growth factor in clinical trials for the treatment of Parkinson’s disease [6, 44, 45]. Our approach would allow for constant expression and secretion of the immunotherapeutic protein within the brain after a single round of administration, eliminating the need for frequent dosing required in current anti-amyloid passive immunotherapy regimens. The efficacy shown here by AAV-scFv-IgG expression in an amyloidosis mouse model demonstrates that this approach holds promise for long-term treatment for a variety of neurodegenerative diseases, particularly as advances in recombinant AAV technology improve the efficiency and feasibility of CNS transduction.

## Materials and methods

### Study design

This study was initiated to design anti-Aβ IgGs for AAV-mediated delivery to the brain for the treatment of Alzheimer’s disease. These IgG constructs were designed and initially tested in vitro 2–4 times to confirm proper expression, assembly and antigen binding activity prior to in vivo experiments. Sample sizes for C57BL/6 or SCID animal studies were set based on variability observed from previous experiments expressing transgenes in vivo using stereotaxic delivery of AAV, and are defined for each experiment. Studies testing in vivo expression were performed 2–3 times. Sample size for the APP mice for amyloid plaque load quantification was set to account for expected inter-animal variability in plaque formation. Based on prior studies using this line [27], the efficacy study was performed once n≥10 per group. Animals were randomly assigned to each group for all studies. ROI identification for automated image analysis was performed by researchers blind to the experimental conditions. All procedures in the USA were performed according to a protocol approved by the Institutional Animal Care and Use Committee at Sanofi US, as per guidelines specified by the Guide for the Care and Use of Laboratory Animals (NIH). Animal studies in France were approved by the French legislation & Sanofi Group Animal Care and Use Committee. Projet CL-N&P-Neurodegeneration/ N° APAFIS 2015112510095869.

### AAV-IgG designs

Variable regions were derived from the anti-Aβ antibody were either from the original 13C3 murine (for AAV-αAβ msIgG) or humanized sequences (for AAV-αAβ IgG) [21] as described in patent applications WO2009/065054 and WO2010/130946, respectively. The huIgG expression vector was generated by inserting the coding sequences for the human IgG4 heavy chain containing two amino-acid substitutions described to reduce half molecules (S241P) and effector functions (L248E) [23] and kappa light chain into the dual promoter cassette shown in Fig 1A. For experiments requiring the mouse IgG1 framework, the original 13C3 antibody [27, 40] was used with the addition of an N297A mutation in the heavy chain to reduce effector function. The AAV-Control IgG vector encoded a huIgG4 PE isotype control antibody that targets a non-mammalian antigen.

### ScFv-IgG design

The design of the scFv-IgG is shown (Fig 4A). Briefly, the variable light and variable heavy chain regions of the parental 13C3 anti-amyloid beta antibody were connected by 3 repeats of a flexible G4S linker to form a vL-vH scFv. The scFv sequence was followed by an additional 9-repeat glycine linker (34) that included the native murine IgG1 hinge and CH2 and CH3 domains to comprise the Fc region of the scFv-IgG. As with the AAV-αAβ msIgG, asparagine 297 of the Fc was mutated to alanine (N297A) to attenuate effector function [30, 46]. A C-terminal 6xHis epitope tag was included to facilitate both in vitro purification and in vivo detection in mice. Expression of the scFv-IgG was driven by an hCMV/hEF1a-promoter expression cassette with a Tbgh polyA.

### Immune tolerance

To induce immune tolerance, APP mice were injected at days 0, 2 and 10 with 7.5mg/kg IP with GK1.5 anti-CD4 monoclonal antibody (Bioxcell). To confirm CD4 T-cell depletion, blood was taken on day 12 by retro orbital sampling into heparin coated tubes. CD4+ T lymphocytes were quantified using FACS analysis on a BD Fortessa using standard protocols with CD45-FITC (clone 104 BD Pharmigen), CD3e-AlexaFluor 647 (clone 17A2, eBioscience) and CD4-PE (RM4-4 clone, BioLegend) antibodies. GK1.5 treated animals had reduced CD4 as evidenced by a ratio of CD4+ lymphocytes/ total CD3+ lymphocytes of 0.04 +/- 0.008 (mean +/-SEM) in the treated mice compared to 0.47 +/- 0.003 from untreated mice.

### Cell culture, protein expression and purification

Expi293 cells (Life Tech) were passaged in Expi293T serum-free medium (Life Tech) and used for protein expression. The expression plasmids were transfected into Expi293 cells via lipid transfection (Fectopro, Polyplus), and the cell culture medium containing secreted protein was collected 4 days later. Following sterile filtration, 6xHis tagged proteins were purified via immobilized metal-affinity chromatography (IMAC). Briefly, proteins were batch adsorbed to cobalt resin (Thermo Scientific) overnight at 4C, washed with 10 column volumes of phosphate buffered saline, then eluted with 500mM imidazole. Proteins were dialyzed into HEPES buffered saline overnight, concentrated (Centricon), and frozen at -80C until use.

### ELISAs

96 well Immulon IIHB (Thermo) plates were either coated with 1 ug/mL Aβ_1–42_ (Bachem H-1368) for the antigen ELISA, or 1 ug/mL mouse anti-huIgG polyclonal Ab (Jackson 209-005-088) to capture total huIgG in carbonate buffer overnight at 25°C. Wells were washed 5X in TBS-0.5% tween (TBST), and blocked in TBSTB (TBST+1.5%BSA) for 1 hour. Standard curves using purified protein were run in parallel with sera or brain homogenates to allow for quantification of bound scFv-IgG or huIgG. Samples were incubated for 2.5hrs, washed 3X in TBST, and then incubated with HRP-conjugated secondary for 1 hour. Following 5x TBST washing, wells were incubated with TMB substrate for 5min before quenching with 0.5M H_2_SO_4_. Plate-bound signal was quantified by absorbance at 450nm (Spectramax M5). All samples were run in triplicate.

### LCMSMS

The LC/MS/MS experiments were carried out on the Q Exactive Mass Spectrometer (Thermo Scientific) coupled with NanoAcQuity LC system (Waters). The IgG from tissue homogenates were specifically enriched and isolated with CaptureSelect HuIgG affinity resins (Thermo Fisher). The enriched IgGs were digested by incubation with trypsin/Lys-C (1:100 w/w) overnight at 37°C after DTT reduction and alkylation. The digestion was terminated by the addition of 1% formic acid (FA). The resulted tryptic peptide mixtures were loaded and separated onto a microcapillary column (75-μm id, 15 cm HSST3, 1.8μm, Waters). Data were acquired in the PRM mode with the resolution of 70,000 (at m/z 200), AGC target 5 × 106, and a 500 ms maximum injection time. The scheduled inclusion list was generated based on the profiling data of the control IgGs. The PRM method employed an isolation of target ions by a 2 Da isolation window, fragmented with normalized collision energy (NCE) of 25. MS/MS scans were acquired with a starting mass range of 100 m/z and acquired as a profile spectrum data type. Precursor and fragment ions were quantified using Skyline (MacCoss Lab Software).

### Surface plasmon resonance

Aβ_1–42_ peptide (Bachem H-1368) was incubated in 10mM HCl at 1 mg/mL overnight at 37°C, shaking at 600rpm. The resulting fibril solution was directly immobilized on a CM5 sensor chip (GE Healthcare) using amine coupling. Antibody or scFv-IgG solutions generated at 50, 30, 20, 10 and 5nM in PBS-+P buffer (GE Healthcare) were injected at relatively high flow rate (50 μL/min) to limit avidity effects. The data were processed using Biacore T200 evaluation software and double referenced by subtraction of the blank surface and buffer-only injection before global fitting of the data to a 1:1 binding model.

### AAV ITR plasmids and adeno-associated viral vector preparation

Expression cassettes for the IgG or the scFv-IgG were subcloned into an AAV2-ITR containing plasmid, with *A1AT* stuffer DNA retained as needed to maintain the AAV genome size for proper packaging. In the case of the dual promoter IgG ITR plasmid, no stuffer DNA was included as the cassette was already the maximum size permitted for efficient packaging. AAV-Empty vector consisted of the CBA promoter, Tbgh polyA, and *A1AT* stuffer DNA. AAV2/1 virus was produced via transient transfection. In brief, HEK293 cells were transfected using PEI (polyethyleneimine) with a 1:1:1 ratio of three plasmids (containing the ITR, AAV rep/cap and Ad helper). The Ad helper plasmid (pHelper) was obtained from Stratagene/Agilent Technologies (Santa Clara, CA). Purification was performed using column chromatography, as previously described [47]. Virus was titered using qPCR against the polyA sequence, and AAVs were stored in 180mM Sodium Chloride, 10mM Sodium Phosphate (5mM Monobasic + 5mM Dibasic), 0.001% F68, pH 7.3 at -80C until use.

### Animals

Animals used were C57BL/6 males obtained from Jackson Labs (Bar Harbor, USA) at 2 months of age unless otherwise specified. Adult SCID mice were obtained from Jackson Labs (B6.CB17-Prkdc^scid^/SzJ) at 2 months of age. The APP transgenic mouse line expresses mutant APP from the Thy1.2 promoter and has been well described and characterized previously [27]. These mice were bred and maintained on a C57BL/6 background at Charles River France facilities until delivery at the indicated ages to Framingham, MA or Chilly-Mazarin, France. Surgical groups were housed singly to enable proper recovery from the brain surgeries. Mice were maintained on a 12-hr light/dark cycle with food and water available ad libitum. Animals were randomized to different groups and analyses were performed with operators blind to the treatment groups.

### Stereotaxic injections

Surgery was performed according to procedures approved by the animal care and use committee. Mice were deeply anesthetized with an intraperitoneal injection of mixture (volume 10mL/Kg): ketamine (100 mg/kg; Imalgene; Merial, France) and xylazine (10 mg/Kg; Rompun; Bayer, France). Before positioning the animal in the stereotaxic frame (Kopf Instruments, USA), the mouse scalp was shaved and disinfected with Vetidine (Vetoquinol, France), a local anesthetic bupivacaine (2mg/kg at a volume of 5ml/kg; Aguettant, France) was injected subcutaneously on the skin of the skull and Emla (Lidocaïne, Astrazeneca) was applied into the ears. During surgery, the eyes were protected from light by vitamin A Dulcis and the body temperature was kept constant at 37°C with a heating blanket.

Samples were injected at a rate of 0.5 microliters per minute. The needle was left in for 2 minutes to prevent flow of sample back through the needle tract, and then slowly raised out of the brain. Unilateral hippocampal injections into APP mice were performed in Chilly-Mazarin, France while unilateral or bilateral injections into all other mice were performed in Framingham, MA. Coordinates for hippocampal injections were: AP -2.0, DV -2.0, and ML +/-1.5. Mice were kept warm and received subcutaneous injection of carprofen (5mg/kg in a volume of 5ml/kg, Rimadyl, Zoetis) following surgery and observed continuously until recovery. At the end of the study, mice were euthanized by anesthetic overdose with euthasol (USA) or ketamine/zylazine (France). Following overdose, mice were kept warm until perfusion with ice-cold PBS.

### Immunohistochemistry

Following perfusion with cold PBS, brain tissue was fixed in 10% neutral buffered formalin (NBF). Formalin fixed tissue was embedded into paraffin, then sectioned at 5μm in the sagittal or coronal plane. All tissue was stained using a Leica BOND RX autostainer. For immunofluorescence staining, heat-mediated antigen retrieval was performed using epitope retrieval solution 1 (ER1; citrate buffer, pH 6.0) for 10 minutes. Tissue was then blocked/permeabilized in goat serum+0.25% triton X-100, then incubated with primary antibodies for 1 hour at RT, washed in TBST, then incubated with secondary antibodies for 30 min. Nuclei were detected using Spectral DAPI (Life). For plaque quantification, tissue was immunostained with biotin-conjugated 4G8 antibody (4G8 clone BioLegend 800701) using the Vectastain ABC (PK-7100) kit as per manufacturer’s instructions without antigen retrieval or formic acid extraction. Pilot experiments validating the 4G8 IHC assay in aged APP/PS1 mice [48] demonstrated that sections treated with or without formic acid exhibited identical levels of extracellular plaque DAB signal (S1 Fig).

### Antibodies

6xHis (Abcam Ab9108, 1:1000 IHC, Invitrogen R931-25, 1:1000 Western, ELISA) GFAP (Ebiosciences, 41-9892-82, 1:200 or Abcam Ab4674, 1:500 IHC) 4G8 (BioLegend 800701, 1:500 IHC). Secondary antibodies from Life Technologies: Cy3 goat anti-mouse, Alexafluor647 goat anti-rabbit, Alexafluor488 goat anti-chicken; all at 1:500. For amyloid DAB: 4G8-biotin (BioLegend 800705 1:250).

### Image analysis

Immunohistochemistry slides were scanned at 20x magnification using Scanscope XT bright-field image scanner (Aperio, Vista, CA) or AxioScanZ1 (Carl Zeiss Microscopy GmBH, Germany). Whole slide images (WSI) of GFAP IHC were viewed and analyzed using HALO image analysis software (Indica Labs, Corrales, NM, USA). For each WSI, the hippocampus region was manually annotated and analyzed for GFAP immunopositive area using HALO’s automated area quantitation algorithm. For each sample, GFAP positive area was divided by the total tissue area for the selected region of interest (ROI) to obtain percent immunopositive area. For plaque analysis, 5μm coronal brain sections were collected from 6 month old APP mice at three different levels, 50μm apart. Cortical and hippocampal ROIs were manually annotated. Amyloid plaque burden was quantified as % DAB+ tissue area using a custom image analysis algorithm developed using ZEN 2 software (Carl Zeiss Microscopy GmBH, Germany). Data were plotted using GraphPad Prism version 6 (GraphPad Software, La Jolla, CA, USA).

### Statistics

Statistical analysis was performed using Graphpad Prism (v6 and v7) using 1-way ANOVA with multiple comparisons (Dunnett) for experiments with more than two groups. Unpaired student’s t test was used for comparison of two groups. *p<0.05, **p<0.01, ***p<0.001. Sample size varied, and is specified for each experiment.

## Supporting information

S1 Fig Comparison of 4G8 IHC with or without formic acid pretreatment in aged APP/PS1 mouse brain. To determine whether formic acid pretreatment was required for amyloid plaque detection using the biotinylated 4G8 antibody (BioLegend), serial FFPE brain sections from mice with preexisting plaque pathology (8 month old APP/PS1) mice were used. For formic acid antigen retrieval, sections were incubated in 80% formic acid in dH2O (pH 1.62 with 5N NaOH) for 4 min at room temperature before proceeding with the IHC protocol described in the main text. Importantly, no difference in beta-amyloid plaque immunostaining was observed between the samples stained with or without formic acid pretreatment (indicated in the top two panels). Demonstrating the specificity of the antibody, no immunostaining was observed in brain sections from a C57Bl/6 mouse (wild type control indicated in lower left). The panel in lower right shows a comparable immunostaining pattern once the IHC protocol was transferred to the BondRX (Leica) automated IHC platform.
(TIF)
