# Supplementary figures and images for: Gene delivery of a modified antibody to Aβ reduces progression of murine Alzheimer’s disease
Source: PLoS One. 2019 Dec 30;14(12):e0226245. doi: 10.1371/journal.pone.0226245 (PMC6936806; doi:10.1371/journal.pone.0226245)

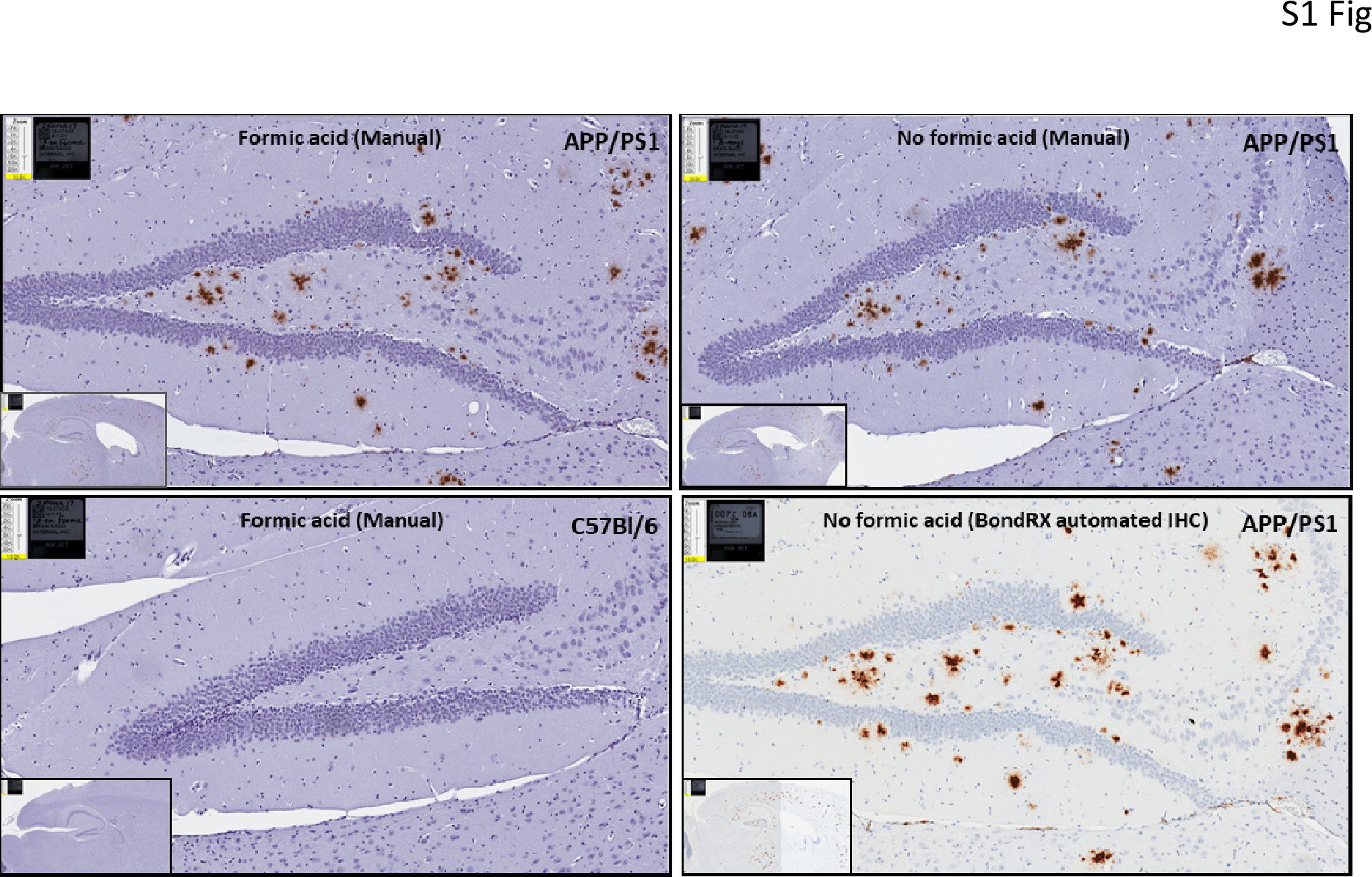

Supplement: S1 Fig — To determine whether formic acid pretreatment was required for amyloid plaque detection using the biotinylated 4G8 antibody (BioLegend), serial FFPE brain sections from mice with preexisting plaque pathology (8 month old APP/PS1) mice were used. For formic acid antigen retrieval, sections were incubated in 80% formic acid in dH2O (pH 1.62 with 5N NaOH) for 4 min at room temperature before proceeding with the IHC protocol described in the main text. Importantly, no difference in beta-amyloid plaque immunostaining was observed between the samples stained with or without formic acid pretreatment (indicated in the top two panels). Demonstrating the specificity of the antibody, no immunostaining was observed in brain sections from a C57Bl/6 mouse (wild type control indicated in lower left). The panel in lower right shows a comparable immunostaining pattern once the IHC protocol was transferred to the BondRX (Leica) automated IHC platform. (TIF) [file pone.0226245.s001.tif]
